# Self-Enhanced Catalytic Activity of Pt/TiO_2_ via Electronic Metal–Support Interaction

**DOI:** 10.1021/acscentsci.2c01546

**Published:** 2023-01-05

**Authors:** Shuheng Tian, Jiarui Li, Dequan Xiao, Ding Ma

**Affiliations:** † Beijing National Laboratory for Molecular Sciences, College of Chemistry and Molecular Engineering Peking University, Beijing 100871, P. R. China; ‡ Center for Integrative Materials Discovery, Department of Chemistry and Chemical and Biomedical Engineering, University of New Haven, West Haven, Connecticut 06516, United States

The metal–support interfacial sites are the main active sites in many heterogeneous catalytic processes. Controlling the metal–support interaction (MSI) is an important method to adjust the property of active metals and improve catalytic efficiency.^1,2^ The classic strong metal–support interaction (SMSI), one of the most common strategies, refers to the coverage of metal nanoparticles by supports, which may inhibit the adsorption of small molecules on metals. Recently, SMSI systems with reducible metal oxide supports have drawn wide attention because of their tunable interfacial sites.^3,4^ In some cases, up to 15-fold enhancement in activity can be achieved by SMSI.^3^ However, SMSI can also cause encapsulation of active metals by the reducible supports, limiting the exposure of interfacial sites and suppressing reaction activities.^5,6^ Thus, constructing an MSI with suitable strength is crucial for maintaining catalytic activity and durability. In a recent issue of *ACS Central Science*, Deng, Wu, Zou, and co-workers reported a supported metal cluster catalyst Pt-mpTiO_2_ with confined Pt nanoclusters.^7^ This catalyst design stabilizes the Pt nanoclusters and introduces a favorable medium MSI effect, providing a substantial amount of Ti^3+^-O_v_-Pt^δ+^interfacial active sites and exhibiting superior catalytic performance compared to traditional Pt/TiO_2_ catalysts.

**Figure 1:**
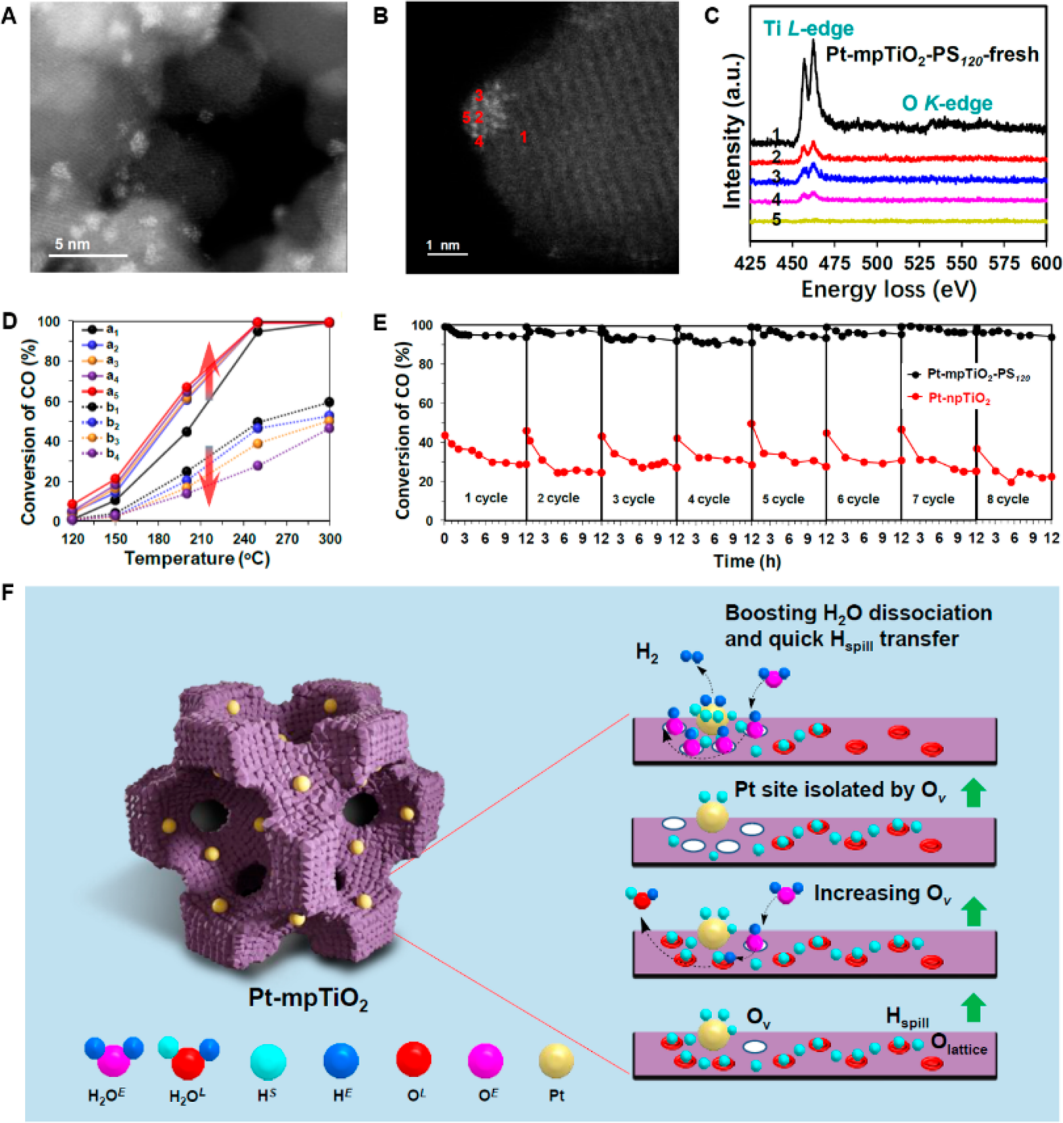
Structure analysis and catalytic performance of the Pt-mpTiO_2_ catalyst. (A, B) Ac-HAADF-STEM image of Pt-mpTiO_2_. (C) EELS spectra of the selected spots in (B). (D) CO conversions as a function of temperature over (a) Pt-mpTiO_2_ and (b) Pt-npTiO_2_ during the cyclic catalytic activity evaluation of five times (reaction conditions: 2% CO, 8% H_2_O, 90% N_2_; WHSV 60,000 mL g_cat_^–1^ h^–1^). (E) Plots of CO conversion to CO_2_ as a function of reaction time at 250 °C over the Pt-mpTiO_2_ and Pt-npTiO_2_ catalysts for the catalytic stability evaluation. (F) Scheme of the mechanism and effect of increasing O_v_ in used Pt-mpTiO_2_. Reproduced with permission from ref (7). Copyright 2022 The Authors. Published by American Chemical Society.

The key factors that affect the construction of proper interfacial sites include the size of the metal nanoclusters and the morphology of the supports. During many reactions, the small active metal nanoclusters tend to aggregate into larger metal nanoclusters that can be easily encapsulated due to SMSI, leading to deactivation of the catalyst. To obtain a favorable medium MSI, mesoporous TiO_2_ (mpTiO_2_) with interconnected uniform mesopores is ideal for carrying Pt nanoclusters. The obtained Pt clusters (1.06 ± 0.06 nm) were distributed evenly in the mesopores of mpTiO_2_, while the hydrophilic pore wall of mpTiO_2_ could inhibit further aggregation of the Pt species (Figure 1A). This geometric structure enhanced the stability of highly dispersed Pt clusters and provided abundant active interfaces. Furthermore, the unique medium MSI was revealed by HAADF-STEM images and EELS spectra (Figure 1B–C), which showed the partial encapsulation of Pt nanoclusters by TiO_*2*_**.** The authors chose the water–gas shift (WGS) reaction, an important industrial reaction, as the model reaction. Compared with the Pt supported on nonporous TiO_2_ (Pt-npTiO_2_), the Pt-mpTiO_2_ catalyst showed increased activity by several fold. Interestingly, an unusual self-enhanced catalytic activity was discovered during the cycling test (Figure 1D,E). The HAADF-STEM and EELS analyses of used Pt-mpTiO_2_ showed that the medium MSI between Pt nanoclusters was preserved, indicating the marvelous stability of Pt-mpTiO_2_.

To understand the mechanism behind the self-enhanced catalytic activity, the authors studied the electronic structure change induced by the MSI. The changes in electronic structure of elements Ti, O, and Pt were shown clearly by the XPS measurements, indicating the formation of Ti^3+^-O_v_-Pt^δ+^ interfacial sites due to the charge transfer between Pt and mpTiO_2_, which can be deemed as electronic MSI (EMSI). The increase of O_v_ concentration in the used Pt-mpTiO_2_ was found by H_2_-TPD and H_2_-TPR, indicating that the *in situ* generated H_2_ spilled over to TiO_2_ and resulted in the reduction of Ti^4+^. During the WGS reaction, the O_v_ played an important role in enhancing the activity (Figure 1F). The initial O_v_ could boost the H_2_O dissociation into H*. Then, the H* activated the lattice oxygen of mpTiO_2_ to generate more O_v_ at Ti^3+^-O_v_-Pt^δ+^ interfacial sites. This catalytic cycle facilitated the activation of H_2_O and the generation of H_2_, indicating that, in addition to promoting the WGS reaction and stabilizing the Pt nanoclusters, this unique Ti^3+^*-*O_v_-Pt^δ+^interfacial structure is also capable of improving the activity and stability of the Pt-mpTiO_2_catalyst in other reactions, demonstrating the generality of this catalytic system.

This work sheds light on a new method to enhance catalytic activity by controlling the MSI effect. It is anticipated that this brand-new strategy will be further applied to other important catalytic reactions, especially those that require active interfacial sites such as CO oxidation, CO_2_ hydrogenation, and WGS reaction. Meanwhile, further research is still needed to better control the MSI effect of heterogeneous catalysts by tuning the geometric and electronic structure.^8^ The interactions can vary greatly between different active metals and supports. Besides the demonstrated characterization technologies in this work, kinetic analysis can be a powerful method to investigate the reaction mechanisms with the MSI effect. For instance, steady-state isotopic transient kinetic analysis (SSITKA)-DRIFTS-mass spectroscopy could give us quantitative information on the dynamic change of the interfacial sites (Ti^3+^-O_v_-Pt^δ+^) *operando*, with an enhanced illustration of the MSI effect.^9^ Despite the considerable challenge of constructing suitable interfacial sites, we expect that more works will be inspired from this work for interfacial catalyst design via MSI in the future.
